# Volatile Composition and Sensory Attributes of Smoothies Based on Pomegranate Juice and Mediterranean Fruit Purées (Fig, Jujube and Quince)

**DOI:** 10.3390/foods9070926

**Published:** 2020-07-14

**Authors:** Hanán Issa-Issa, Marina Cano-Lamadrid, Ángel Calín-Sánchez, Aneta Wojdyło, Ángel. A. Carbonell-Barrachina

**Affiliations:** 1 Department of Agro-Food Technology, Universidad Miguel Hernández de Elche (UMH), Escuela Politécnica Superior de Orihuela (EPSO), Research Group “Food Quality and Safety”, Ctra. Beniel, km 3.2, 03312 Orihuela, Alicante, Spain; hissa@umh.es (H.I.-I.); marina.cano.umh@gmail.com (M.C.-L.); angel.carbonell@umh.es (Á.A.C.-B.); 2 Department of Fruit, Vegetable and Plant Nutraceutical Technology, Wrocław University of Environmental and Life Sciences, 37 Chełmońskiego Street, 51-630 Wrocław, Poland; aneta.wojdylo@upwr.edu.pl

**Keywords:** *Punica granatum*, *Ficus carica*, *Ziziphus jujuba*, *Cydonia oblonga*, descriptive sensory analysis, volatile profile

## Abstract

To increase the intake of fruits and vegetables—especially among young people—the food industry is trying to develop new, easy-to-eat and long-shelf-life products, such as smoothies. Nowadays, consumers are choosing their foods based not only on nutritional/functional properties (content of polyphenols, vitamins, minerals, among others), but also on sensory attributes. The aim of this study was to investigate the volatile composition by HS-SPME and the sensory profile by descriptive sensory analysis of novel smoothies prepared by blending fig, jujube or quince purée with pomegranate juices (cv. *Mollar de Elche* or *Wonderful*) at two ratios purée:juice (40:60 or 60:40). Twenty-three volatile compounds were identified by GC-MS and classified as alcohols, aldehydes, esters, furans, ketones, terpenes and terpenoids. Among volatile compounds, the five predominant ones in the studied smoothies were: (i) 5-HMF (30.6%); (ii) 3-hexen-1-ol (9.87%); (iii) hexanal (9.43%); (iv) 1-hexanol (8.54%); and (v) 3-octanone (7.67%). Fig smoothies were sweet and had flavor and volatiles related to fig, pomegranate, and grape. While jujube products were bitter and had jujube and pear notes. Finally, quince smoothies were consistent, sour and had quince, apple and floral notes. Thus, the type of fruit purée used clearly determined the flavor of the final product. The smoothies prepared with *Mollar de Elche* pomegranate juice were characterized by having high intensity of pear odor/aroma and consistency, and the *Wonderful* smoothies were characterized by lower consistency and more intense pomegranate aroma and sourness.

## 1. Introduction

Nutrition is the most important external factor influencing children’s development. Its influence is essential from birth through childhood. Consumption of fruit and vegetables is important as it plays an essential role in preventing childhood obesity, and preventing many diseases, including certain cancers, osteoporosis, diabetes, coronary heart disease, stroke, neuronal degeneration, and type II diabetes [1,2,3]. Therefore, the World Health Organization (WHO) recommends eating a minimum of 400 g of fruits and vegetables per day to improve health and prevent the above-mentioned chronic diseases. Because current consumption is lower than the recommended intake, the development of easy-to-eat fruit-based products such as smoothies could be a good option. Despite of the natural sugar content, smoothies could reach the promotion into the children’s diet [4,5]. Smoothies are beverages containing a blend of fruit pulp, fruit juice, ice, yoghurt, and/or milk. They are becoming a so popular way to consume fruits, especially among young people. These products are typically purchased freshly prepared from juice bars or as a processed product (mildly pasteurized) from the refrigerated section of retail outlets. Even after the economic crisis of 2007–2008, smoothies remained a popular and convenient way of consuming fruit [6]. Fruit components of smoothies could be considered as natural foods because of their nutrient profile or health-protecting qualities [7].

The southeastern part of Spain is one of the most intensively Mediterranean agricultural areas dominated by fruit orchards and vegetables fields. Some of these crops grown in this area, include fruits with appropriate characteristics for developing new products (organoleptic and functional properties), but underutilized, such as figs (*Ficus carica*), jujubes (*Ziziphus jujuba*) and quinces (*Cydonia oblonga*). It was recently found that the blend of pomegranate juice with the above mentioned fruits purée seemed a great opportunity to promote their use in an easy, sustainable and healthy way [5]. A positive effect of the addition of fig, jujube, and quince purée was observed in the nutritional and functionality of the novel pomegranate smoothies. For example, the addition of jujube contributed to an enrichment of the final smoothies in terms of vitamin C and organic acid content, while an increase of pectin content was found in fig and quince pomegranate-based smoothies [5].

On the other hand, the high interest in consuming fruit and vegetable products clearly shows that consumers are choosing products based not only on nutritional/functional properties (content of polyphenols, vitamins, minerals, among others), but also on sensory attributes (taste, smell, appearance, or even satisfaction) [8]. For this reason, fruit smoothies have become popular among health-conscious consumers and are among the major sources of bioactive compounds in daily diet [9,10]. Therefore, after knowing the nutritional and functional quality of the smoothies previously developed, the next steps should be to evaluate their volatile compositions and sensory profiles.

Taking all above mentioned into account, the aim of this study was to study the volatile composition and sensory profile of 12 different smoothies prepared using pomegranate juice (from 2 cultivars, cv., *Mollar de Elche* and *Wonderful*) and purée of Mediterranean crops (figs, jujubes and quinces) at different ratios purée:juice.

## 2. Materials and Methods

### 2.1. Plant Material

Pomegranates (*Punica granatum*) cv. *Mollar de Elche* and *Wonderful*, figs (*Ficus carica*) cv. *Colar*, jujubes (*Ziziphus jujuba*), cv. *Grandes de Albatera* and quinces (*Cydonia oblonga*) cv. *Gigante de Vranja* were hand-harvested in between mid-August and mid-October 2016 at a commercial maturity stage. The different stages of the smoothie preparation process as well as the ratio of purée:juice were previously described by Cano-Lamadrid et al. [5]. Briefly, the stages of the smoothie preparation process were:

- Purée preparation: figs (F), jujubes (J), or quinces (Q) were peeled, ground, and heated at 80 °C in a Thermomix device (Vorwerk, Wuppertal, Germany); 10 mL of rhubarb juice per 1 kg of fruit were added to prevent enzymatic browning of the fruit due to the high oxalic acid concentration which chelates copper from the active site of polyphenol oxidase (PPO) [11,12]. After, the particle size of the mixture was reduced in a blender (Symbio, Zelmer, Rzeszów, Poland) until getting a thin purée. Then, the samples were cooled to room temperature.
- Pomegranate juice preparation: pomegranate fruits (*Mollar de Elche* and *Wonderful*, PM and PW, respectively) were cut in halves, and arils were manually separated from the husk and ground in a Thermomix to obtain the pomegranate juices.
- Partial products in appropriate proportions preparation: purée and juices samples, immediately after their preparation, were mixed in the proportions 40:60 (40F:60 PM; 40F:60PW; 40J:60 PM; 40J:60PW; 40Q:60 PM; 40Q:60PW) and 60:40 (60F:40 PM; 60F:40PW; 60J:40 PM; 60J:40PW; 60Q:40 PM; 60Q:40PW), obtaining 12 samples. Then, the products were heated to 100 °C, put into glass jars (130 mL) and pasteurized (10 min at 90 °C).

### 2.2. Volatile Compounds

Volatile composition of the samples under analysis was obtained by headspace solid phase microextraction (HS-SPME). Five g of each smoothie + 10 mL ultrapure water were placed into 50-mL vials with polypropylene caps and PTFE/silicone septa. A magnetic stirring bar was added, together with NaCl (15%) and the vial was placed in a water bath with controlled temperature and automatic stirring. A 50/30 μm DVB/CAR/PDMS fiber (Supelco, Bellefonte, PA, USA) (high capacity of trapping fruit volatile compounds) was exposed to the sample headspace for 50 min at 40 °C to simulate the mouth temperature during the chewing process. Desorption of the volatile compounds from the fiber coating was carried out in the injection port of the GC-MS for 3 min.

The identification and semiquantification of the volatile compounds was performed on a gas chromatograph (GC-MS), Shimadzu GC-17A (Shimadzu Corporation, Kyoto, Japan), coupled with a Shimadzu mass spectrometer detector GC-MS QP-5050A. The chromatographic set up and conditions were identical to those recently reported, with the only exception that the column used was a Restek Rxi-1301 Sil MS (Restek Corporation, Palo Alto, USA) of 30 m × 0.25 mm internal diameter, 0.25-μm film thickness. Analyses were carried out using helium as carrier gas at a flow rate of 6 mL min^−1^ in a split ratio of 6 and a program: (a) initial temperature 80 °C; (b) rate of 3.0 °C min^−1^ to 210 °C and hold for 1 min; (c) rate of 25 °C min^−1^ from 210 to 300 °C and hold for 3 min. Injector and detector temperatures were held at 230 and 300 °C, respectively.

Most of the compounds were simultaneously identified by using 3 different analytical methods: (1) retention indices, (2) GC-MS retention times (authentic chemicals), and (3) mass spectra (authentic chemicals and Wiley spectral library collection). Identification was considered tentative when it was based only on mass spectral data. The volatile composition analysis was run in triplicate and results were expressed as percentage of the total area represented by each one of the volatile compounds.

### 2.3. Sensory Evaluation with Trained Panel

Eight trained panelists (aged 30 to 55 years; 4 females and 4 males) from the *Escuela Politécnica Superior de Orihuela* (EPSO), *Universidad Miguel Hernández de Elche* (UMH) with more than 500 h of training in sensory testing participated in this study. The panel was selected and trained following the ISO standard 8586-1 (1993), and it is specialized in descriptive sensory evaluation of pomegranate products [13,14,15,16]. For the present study, the panel worked during 2 orientation sessions (90 min for each one) discussing the main organoleptic characteristics of commercial smoothies and fruit-based: pomegranate, figs, jujube, and quinces. The lexicon used for describing the flavor and texture attributes was based on the previously developed by other authors [17,18]. Both lexicons were adapted for smoothies based on pomegranate during the orientation sessions (Table 1). Samples were served into odor-free, disposable 90 mL covered plastic cups, at room temperature and were coded using 3 digit numbers as previous studies indicated [15]. Unsalted crackers and distilled water were provided to panelists to clean their palates between samples. The panel used a continuous numeric scale (0–10) for quantifying the intensity of smoothie attributes, where 0 represents none and 10 extremely strong, with 0.5-unit increments.

### 2.4. Statistical Analysis

Data were subjected to analysis of variance (ANOVA), after checking the normality and homogeneity of the variance, and later to Tukey’s multiple-range test to compare the means. Differences were considered statistically significant at *p* < 0.05. All statistical analyses were performed using Statgraphics Plus 5.0 software (Manugistics, Inc., Rockville, MD, USA). Instrumental parameters correlated with sensory descriptors were used to perform a principal component analysis (PCA regression map) and a dendrogram analysis using XLSTAT Premium 2016 (Microsoft Corporation, Redmond, WA, USA). Euclidean distance by Ward method was performed for the dendrograms of clusters.

## 3. Results and Discussion

### 3.1. Volatile Profile and Composition

HS-SPME is a standard method used for the isolation of volatile compounds; and it is considered as an environmentally friendly technique (due to no solvents are used), selective and very sensitive [19]. This technique has been successfully used to establish the volatile profiles of different matrices such as herbs, wines, vegetables, and fruits [20]. Volatile composition has been investigated in different pomegranate products, such as pomegranate juice [21,22] and dehydrated pomegranate arils [16]. It was also studied in different fruits such as jujube [23], quinces [24] and figs [25]. However, the present study is the first one evaluating the combined effects of two factors (pomegranate cultivar and ratio purée:juice) on the volatile profile of smoothies blended with different Mediterranean fruits. Table 1 shows the retention indices used for the identification of the compounds, together with the main sensory descriptors of each of the volatiles. Twenty-three volatile compounds were isolated, identified, and their relative abundance determined in the pomegranate smoothies blended with fig, jujube and quince purée samples using this method. Previously, 12 and 14 different compounds were identified in the PW and PM juices [22], indicating that the addition of Mediterranean fruits increased the volatile profile on pomegranate products. Among identified volatile compounds (Table 2), several common compounds were previously detected in *Mollar de Elche* and *Wonderful* pomegranate juices (V1, V3, V4, V7, V11, V13, V15, V17, V18, V19 and V20) [22], and in heat treated pomegranate-based products (dried arils) (V1, V3, V4, V13, V17, V18 and V21) [16]. On the other hand, V4, V6, V11, V12, V13, V14, V15, V18 and V19 were previously identified in quinces fruits [24], while V1, V3, V4, V8, V11, V12,V14, V14, V17 and V19 were identified in previous studies of jujube fruits [23,26,27]. As to figs fruits and dried figs, V1, V2, V4, V5, V6, V8, V10, V11, V13, V14, V15, V16 and V18 were already detected [25,28]. The combinations of different fruit matrix and heat treatments for pasteurization could generate other volatile compounds not previously described and identified (V9, V22 and V23).

Eleven compounds were common in all 3 types of smoothies (fig, jujube, and quince), including for instance hexanal, furfural, 3-heptanone, hexyl acetate, linalool and HMF (Table 2).

At the beginning, statistics were preformed individually for each type of smoothie because of the completely different nature of the products under study, and later, the effect of purée fruit was also analyzed. Table 3 shows the relative abundance of the volatile compounds, grouped by chemical family, in the smoothies prepared with the 2 pomegranate cultivars (*Mollar de Elche* and *Wonderful*) and 3 fruits purée (figs, jujubes and quinces) for 2 ratios purée:juice (40:60 and 60:40). To make the discussion of this section easier, the pomegranate volatile compounds have been grouped into 7 chemical families:

- Alcohols (ALCs): 3-hexen-1-ol (V3), 1-hexanol (V4), 1-octanol (V14), and 2-ethyl-1-hexanol (V15);
- Aldehydes (ALDs, total aldehydes): hexanal (V1), 2-heptenal (V8), octanal (V11), and nonanal (V18);
- Esters (ESTs): hexyl acetate (V12), and ethyl octanoate (V19);
- Furans (FURs): furfural (V2), and 5-HMF (V21);
- Ketones (KETs): 3-heptanone (V5), 3-octanone (V9), and β-damascenone (V22);
- Terpenes (TEs): α-pinene (V6), β-pinene (V7), α-terpinene (V10), limonene (V13) and α-gurjunene (V23);
- Terpenoids (TOs): linalool oxide (V16), linalool (V17), and terpinen-4-ol (V20).

The main chemical groups of the pomegranate smoothies were: (i) furans, representing (32.8% ± 6.8%) of the total concentration of aroma compounds, followed by (ii) aldehydes (20.2% ± 4.0%), (iii) alcohols (19.3% ± 3.8%), (iv) ketones (10.8% ± 4.4%), (v) terpenoids (6.6% ± 2.7%), (vi) terpenes (5.9% ± 1.2%), and (vii) esters (4.6% ± 1.4%). It can be observed that volatile profile differed between the fresh pomegranate juices in which ALCs were the predominant family (67%, mainly 1-hexanol and 3-hexen-1-ol) in PW juices, while ALDs (30%) played an important role and were the most abundant chemical family in the PM juices [22].

The 5 predominant compounds in the studied smoothies were: (i) 5-HMF (mean for all samples 30.6%); (ii) 3-hexen-1-ol (9.87%); (iii) hexanal (9.43%); (iv) 1-hexanol (8.54%); and (v) 3-octanone (7.67%). The fact that the predominant compound was 5-HMF was unexpected. The furanic compound 5-HMF forms as an intermediate in the Maillard reaction between hexoses and amino components, and from direct dehydration of sugars under acidic conditions (caramelization) during thermal treatments applied to foods [29]. For instance, this compound is used as an indicator of the intensity of thermal treatment in honey [30]. In a previous study, 5-HMF and furfural were even found in the optimized dehydrated pomegranate arils [16]. However, the novel pomegranate smoothies highlighted by having less content than the above mentioned product. Without any doubt this compound is generated during the two heating steps of the smoothie preparation. The other 4 compounds are typical of fruits and fruit-based products; for instance, 3-hexen-1-ol, hexanal and 1-hexanol are key compounds of the peach flavor [31]. As 3-octanone was a predominant compound in the volatile composition of fresh wild mushrooms [32].

Hexanal, 3-hexen-1-ol and 3-octanone were more abundant in *Mollar de Elche* samples, while linalool and 5-HMF predominated in *Wonderful* smoothies (Table 3). It is worth mentioning that certain compounds can be used as an indicator of the fruit purée used in the smoothies. For instance, the volatile compounds exclusively identified in figs smoothies were: α-pinene, 1-octanol, ethyl octanoate, terpinen-4-ol and α-gurjunene. On the other hand, linalool oxide was exclusively present in the jujube smoothies. Quinces did not provide any exclusive compound to the smoothies. Formulation with a higher percentage of pomegranate juice (60%) led to higher abundance of 3-hexen-1-ol and linalool, while 60% of fruit purée increased the content of 3-octanone (Table 3).

As a brief summary of this section, it can be stated that *Mollar de Elche* pomegranate juice and smoothies prepared using fig and jujube purées were less sensitive to heat treatment than *Wonderful* and quince smoothies, as reflected by lower 5-HMF contents.

Recently, consumers’ overall liking (“drivers of liking”) of pomegranate-based products (dehydrated arils) was positively linked with the presence of aldehydes, esters, aliphatic alcohols and terpenes [16] which were presented in the novel developed products. Industry could use these liking drivers as quality indicators for improving their commercial and future novel smoothies.

### 3.2. Descriptive Sensory Analysis

An appropriate performance of the panel was observed with a good reproducibility by the end of the orientation sessions. Sixteen attributes (odor, basic tastes, flavor, texture, and defects) were used to fully describe the smoothies and are presented in Table 4. The smoothies prepared with *Mollar de Elche* pomegranate juice presented higher intensity of the fruit purée (F, J or Q) and pear odor, while the *Wonderful* smoothies had more intense pomegranate odor. The use of figs intensified the grape and pomegranate odor notes, while jujubes increased the pear notes. Regarding the basic tastes, *Mollar de Elche* pomegranate smoothies led to sweeter notes compared to the scores of *Wonderful* which were defined as sourer samples. With respect to the fruit purée, the use of figs intensified the sweetness (Table 4).

Regarding to flavor attributes, similar trends as odor attributes was observed. *Wonderful* samples led to higher scores of pomegranate compared to *Mollar de Elche* smoothies that presented higher intensities of fruit purée (F, J or Q). In accordance with our results, previous study indicated that the use of *Wonderful* juices in pomegranate products enhanced pomegranate notes [16].

The fiberness and consistency were the texture attributes evaluated and highly significant differences were observed, especially in the product fiberness. The use of jujubes and quinces led to the highest intensities of fiberness and consistency, respectively. Therefore, the type of fruit purée used had an important effect on fiberness, with jujubes leading to the highest intensity and quinces to intermediate values. Finally, it is important to highlight that the factor having the lowest influence on the sensory profile was the ratio purée:juice, which only influenced the consistency (with the 60:40 products being the most consistent ones). Recently, it was observed that the pectin content increased when using the highest content of fruit purée in smoothies [5]. Pectin has techno–functional characteristics which enhance texture of the smoothies by the reaction of certain water-soluble pectic substances with Ca ions to form some Ca pectates [5]. The purée:juice ratio can, therefore, be adjusted accordingly to the consistency preferred by the consumers. The price of the fruits used for the purée should be also be taken into consideration in order to produce a smoothie with high qualitative characteristics, but at affordable price.

It is worth mentioning that a previous study indicated that consumer’s overall liking in a jujube fruit consumer study was highly correlated with jujube flavor (high intensity), sweetness (high intensity), and bitterness (low intensity) [23]. In our study, the highest jujube notes and sweetness was found for the ratio 40J:60 PM. It represents a good starting point for further development/exploitation of novel smoothies. Similar trend was found in dried pomegranate arils, where the consumer´s overall liking was linked with fresh pomegranate flavor [16].

### 3.3. Principal Component Analysis and Pearson’s Correlations

**Figure 1:**
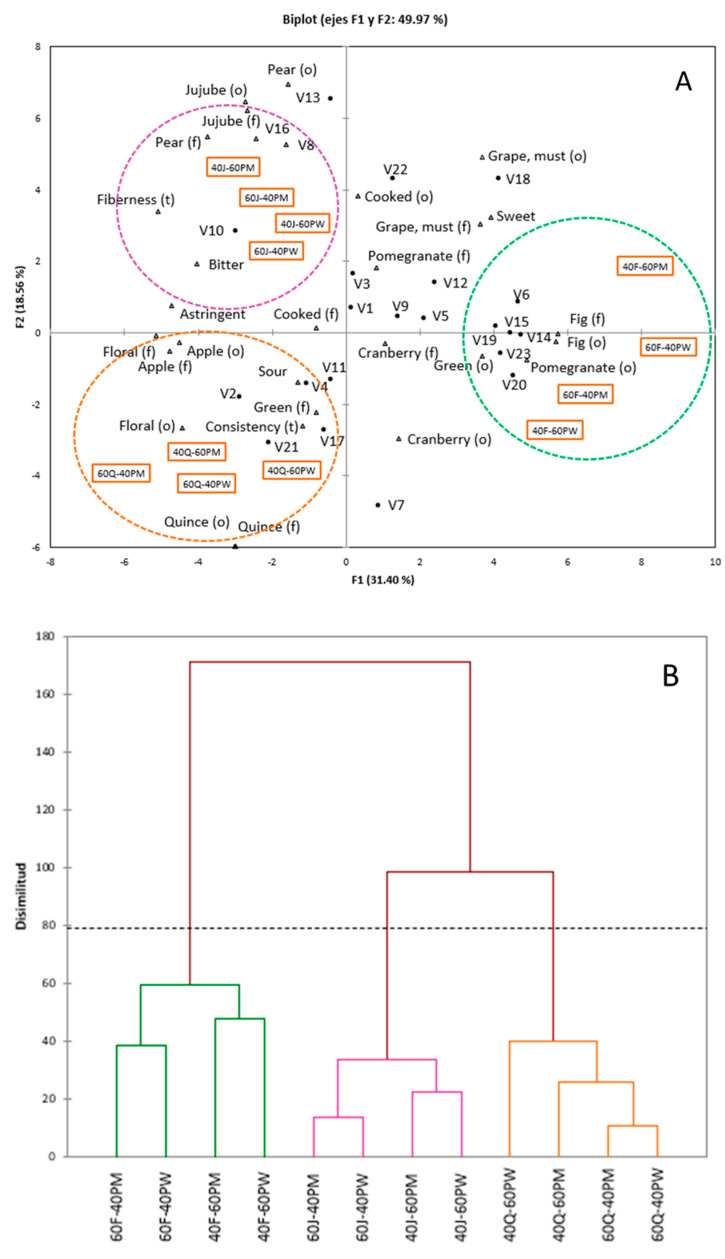
(**A**) PCA and (**B**) cluster maps prepared using (•) volatile compounds and (Δ) sensory profile of smoothies prepared by blending pomegranate juice (PM and PW for “*Mollar de Elche*” and “*Wonderful*”, respectively) with fig (F), jujube (J), or quince (Q) purée.

A principal component analysis (PCA) was conducted to clearly see the relationships among the 12 smoothies, their volatile composition and sensory profile. Figure 1 shows that the first principal component (F1) explained 31.40% of the total data variance and the second one (F2) explained 18.56% of the total variance.

As can be seen, samples were grouped mainly according to the type of fruit purée in the smoothies, regardless of the purée:juice ratio and the pomegranate cultivar. Figure 1B shows how samples are grouped into 3 main clusters. Fig smoothies were characterized by high intensity of fig, pomegranate, cranberry, green, grape notes and sweetness, and with the following volatile compounds: V6, V14, V15, V19 V20, and V23, which present the following odor descriptors: woody, floral, citrus, and green. The relationship between volatile compounds and fig odor and aroma were backed up by significant values of the Pearson’s correlations (Table 4). Jujube smoothies were characterized by high intensity of jujube and pear notes, fiberness and bitterness, and were associated with V8, V10, V13, and V16, being citrus and floral the main descriptors. Values above 0.7 for Pearson’s correlations were found among jujube and these volatile compounds (Table 4). Finally, quince smoothies were characterized by high intensity of consistency and quince, green, apple and floral notes, and sourness, and were linked with V2, V4, V17 and V21. The descriptors of these volatiles are caramel, woody and floral.

Pearson’s correlation coefficient (Table 5) showed that fig odor and fig flavor was positively correlated (R > 0.6; *p*-value < 0.05) with V6, V14, V15, V18, V19, V20, V23. No significant (*p*-value >0.05) correlation was observed among pomegranate flavor with volatile compounds, while pomegranate odor was well-correlated with V20 and V23 (R > 0.7; *p*-value < 0.05). In addition, no significant correlation between volatile compounds and cranberry aroma, cranberry odor, green aroma, and sour was found. As to jujube odor and flavor, Pearson’s coefficient showed that was positively correlated (R > 0.6; *p*-value < 0.05) with V8, V13 and V16. Quince odor and flavor was not positively correlated with any identified volatile compound. Pear odor and flavor, and grape odor and flavor were positively correlated with V8, V13 and V16, and V6, V14, V18 and V23, respectively (R > 0.6; *p*-value < 0.05). Sweet was the highest correlated basic taste with V6, V18, V22 and V23 (R > 0.6; *p*-value < 0.05).

## 4. Conclusions

The volatile composition and the sensory profile of novel smoothies prepared blending fig, jujube or quince purée with pomegranates juices (cv. *Mollar de Elche* or *Wonderful*) at two ratios of purée:juice (40:60 or 60:40) were studied. Twenty-three volatile compounds were identified, the five predominant ones: (i) 5-HMF (mean for all samples 30.6%); (ii) 3-hexen-1-ol (9.87%); (iii) hexanal (9.43%); (iv) 1-hexanol (8.54%); and (v) 3-octanone (7.67%). Fig smoothies were sweet and had flavor and volatiles related to fig, pomegranate, and grape. Meanwhile, jujube products were bitter and had jujube and pear notes. Finally, quince smoothies were sour and had quince, apple and floral notes. Thus, the type of fruit used clearly determined the flavor of the final product. The smoothies prepared with *Mollar de Elche* pomegranate juice were characterized by having high intensity of pear odor/aroma and consistency. While *Wonderful* smoothies were characterized by lower consistency and more intense pomegranate aroma and sour. However, further research is still needed to fully optimize these novel products. Two ways of improvement can be researched: (i) increasing pomegranate notes; and (ii) avoiding undesirable compounds after the Maillard Reaction. After knowing the volatile compounds and sensory profiles of these developed smoothies, it is worth to continue researching in this area studying the volatile composition of other types of smoothies and other types of pasteurization treatments to avoid undesirable compounds and aromas. To know the active odor compounds of these products also can be necessary in the future studies. Moreover, consumer studies should be carried out to know the drivers of smoothie’s consumer acceptance.

## Figures and Tables

**Table 1:** Sensory descriptors for odor, flavor, basic tastes and defects.

Descriptor	Definition	References
Odor/Flavor attributes		
Pomegranate	Sweet and fruity flavor associated with pomegranate	Freshly harvested pomegranate at optimum maturity index, cv. *Wonderful* = 8.0
		Freshly harvested pomegranate at optimum maturity index, cv. *Mollar de Elche* = 5.0
Fig	Sweet and fruity flavor associated with figs	Freshly harvested fig, cv. *Colar* = 9.0
Jujube	Sweet and fruity flavor associated with jujube	Freshly picked jujubes at best picking time, cv. *Grande de Albatera* = 9.0
Quince	A floral, fresh, and fruity aromatics associated with quince	Freshly harvested quinces, cv. *Vranja* = 6.5
Apple	Aromatic compounds associated with processed apple juice and cooked apples	Hacendado mango–apple nectar = 5.5
Pear	Sweet, slightly musty, floral, honey/caramel-like, fruity aromatic associated with ripe pears	Hacendado pear nectar = 6.5
Grape must	Sweet and fruity aromatics from fresh grapes	Grape juice (Welch’s Concord) = 10
Cranberry	Aromatic associated with cranberries	Fresh cranberries = 10
Floral	Sweet, heavy aromatics blend of a combination of flowers	Geraniol (1000 mg L^−1^) = 4.0
Green	Green, fresh aromatics associated with green vegetables and newly cut vines and stems; related to cucumber	Trans-2-hexen-1-ol 5000 ppm = 4.0
		Heinz tomato ketchup (vinegar) = 4.5
		Freshly sliced tomatoes = 10.0
Basic tastes		
Sweet	The fundamental factor associated with a sucrose solution	3% sucrose solution = 2.0
		6% sucrose solution = 4.0
		12% sucrose solution = 8.0
Sour	The taste factor associated with some organic acid, specifically citric acid	0.043% citric acid solution = 2.0
		0.064% citric acid solution = 3.0
		0.120% citric acid solution = 5.0
		0.168% citric acid solution = 7.0
Bitter	The taste factor associated with a caffeine or quinine solution	0.008% caffeine solution = 1.0
		0.15% citric acid solution = 2.0
Astringent	Dry sensation on the surface of the tongue or mouth associated with alum solution	0.03% alum solution = 1.5
		0.05% alum solution = 2.5
		0.1% alum solution = 5.0
Texture		
Fiberness	Geometric property of the texture linked with the perception of the shape and orientation of the particles in the product	Fresh jujube purée (100%) = 9
		Diluted jujube purée (1:1) = 4.5
Consistency	The force required to move the product across the tongue	Distilled water = 1
		Condensed milk = 10
Defect		
Cooked	Reminiscent aromatic compounds of fruit and/or vegetables after heating	Frozen orange concentrate (Minute Maid)-reconstituted = 4

**Table 2:** Retention indices and sensory descriptors of the volatile compounds in smoothies prepared by blending pomegranate juice with fig, jujube, or quince purée.

Code	Compounds ^‡^	Material ^γ^	RT (min)	Retention Indexes ^†^		Descriptors ^‡^
				Exp	Lit	
V1	hexanal	F, J, Q	6.76	830	835	Fatty, green
V2	furfural	F, J, Q	8.58	894	899	Almond, woody
V3	3-hexen-1-ol	F, J, Q	8.85	902	905	Banana
V4	1-hexanol	F, J, Q	9.11	909	912	Green, herbaceous
V5	3-heptanone	F, J, Q	9.81	927	923	Green, fruity, fatty
V6	α-pinene	F	10.13	935	937	Woody
V7	β-pinene	F, Q	12.47	995	998	Woody
V8	2-heptenal	F, J, Q	12.99	1007	904	Apple, lemon, green, spicy
V9	3-octanone	F, J, Q	13.33	1015	1024	Banana, berry, spicy, green
V10	α-terpinene	J, Q	14.12	1032	1034	Berry, lemon, vegetable
V11	octanal	F, J, Q	14.52	1042	1029	Honey, fruity, fatty, citrus
V12	hexyl acetate	F, J, Q	14.57	1042	1042	Apple, cherry, floral, pear
V13	limonene	F, J	14.75	1046	1046	Lemon, orange, citrus
V14	1-octanol	F	15.75	1068	1123	Citrus, fatty, woody
V15	2-ethyl-1-hexanol	F, Q	15.83	1069	1070	Oily, rose, sweet
V16	linalool oxide	J	17.82	1112	1114	Floral
V17	linalool	F, J, Q	19.08	1138	1142	Lemon, orange, floral
V18	nonanal	F, J	19.29	1142	1154	Apple, coconut, grape
V19	ethyl octanoate	F	22.95	1218	1231	Apricot, floral, pear
V20	terpinen-4-ol	F	23.18	1222	1226	Citrus, woody, herbaceous
V21	5-HM	F, J, Q	28.84	1340	1362	Butter, caramel, musty
V22	β-damascenone	F, J	33.46	1438	1459	Apple, herbaceous, woody
V23	α-gurjunene	F	33.64	1442	1436	Woody

**^γ^** F = fig; J = jujube; Q = quince; ^†^ RT = retention time; Exp = experimental; Lit = literature. ^‡^ National Institute of Standards and Technology, NIST (2020); SAFC (2012).

**Table 3:** Relative abundance (%) of volatile compounds in smoothies prepared by blending pomegranate juice with fig, jujube, or quince purée.

ANOVA (AN) ^†^		Fig Smoothies				AN	Jujube Smoothies				AN	Quinces Smoothies							
		40F:60 PM	60F:40 PM	40F:60PW	60F:40PW		40J:60 PM	60J:40 PM	40J:60PW	60J:40PW		40Q:60 PM	60Q:40 PM	40Q:60PW	60Q:40PW	AN	F	J	Q
ALCs																			
V3	***	25.1 ± 5.02a ^‡^	0.46 ± 0.09c	6.80 ± 1.36b	3.19 ± 0.64bc	***	13.0 ± 3.73a	11.6 ± 0.94a	4.71 ± 2.33b	12.7 ± 6.05a	***	23.6 ± 9.75a	2.28 ± 0.80c	4.02 ± 0.32c	10.9 ± 2.17b	NS	8.89 ± 11.1	10.5 ± 3.91	10.2 ± 9.66
V4	***	13.7 ± 2.74a	nd	8.38 ± 1.68b	4.48 ± 0.90bc	**	9.34 ± 2.32b	5.73 ± 0.79bc	3.94 ± 1.15c	10.8 ± 2.17a	***	18.2 ± 6.19a	7.56 ± 1.02b	5.12 ± 1.07b	15.2 ± 3.05a	NS	8.85 ± 4.62	7.46 ± 3.18	11.5 ± 6.20
V14	NS	1.02 ± 0.20	0.84 ± 0.17	0.66 ± 0.13	nd ^ϒ^		nd	nd	nd	nd		nd	nd	nd	nd	–	–	–	–
V15	*	4.14 ± 0.83a	0.87 ± 0.17b	0.56 ± 0.11b	0.72 ± 0.14b		nd	nd	nd	nd	NS	0.44 ± 0.09	nd	0.38 ± 0.08	0.56 ± 0.11	NS	1.57 ± 1.72	–	0.34 ± 0.24
∑ALCs		44.0	2.17	16.4	8.39		22.3	17.3	8.65	23.5		42.24	9.84	9.52	26.7				
ALDs																			
V1	***	19.0 ± 3.80a	2.23 ± 0.45c	3.81 ± 0.76c	8.41 ± 1.68b	***	3.89 ± 1.52c	19.7 ± 3.73a	5.32 ± 0.86c	10.9 ± 1.98b	***	29.8 ± 13.7a	1.06 ± 0.15c	3.09 ± 0.62bc	5.91 ± 1.18b	NS	8.36 ± 7.55	8.94 ± 7.15	9.96 ± 13.4
V8	*	3.88 ± 0.78a	1.05 ± 0.21b	1.24 ± 0.25b	1.49 ± 0.30b	***	4.55 ± 2.74c	19.5 ± 3.90a	7.31 ± 1.46b	19.1 ± 3.83a	NS	0.37 ± 0.04	nd	0.27 ± 0.05	0.32 ± 0.06	*	1.91 ± 1.32b	12.6 ± 7.82a	0.24 ± 0.16c
V11	**	2.66 ± 0.53ab	5.63 ± 1.13a	1.13 ± 0.23b	1.66 ± 0.33b	*	1.06 ± 1.42b	4.69 ± 0.94a	2.84 ± 0.57b	3.60 ± 0.72ab	**	0.55 ± 0.11b	0.22 ± 0.03b	18.3 ± 3.66a	0.61 ± 0.12b	NS	2.77 ± 2.01	3.05 ± 1.53	4.92 ± 8.93
V18	**	5.97 ± 1.19a	1.80 ± 0.36b	3.45 ± 0.69ab	4.75 ± 0.95a	NS	1.45 ± 1.60	3.48 ± 0.67	3.45 ± 0.67	2.60 ± 0.50		nd	nd	nd	nd	NS	3.99 ± 1.79	2.65 ± 0.93	–
∑ALDs		31.5	10.7	9.63	16.3		11.0	47.4	18.9	36.2		30.7	1.28	21.7	6.84				
ESTs																			
V12	***	4.22 ± 0.84b	3.26 ± 0.65b	2.21 ± 0.44b	16.2 ± 3.23a	**	1.82 ± 4.74b	3.37 ± 0.67b	8.20 ± 1.64a	2.87 ± 0.57b	**	3.30 ± 0.66b	nd	4.68 ± 0.94a	0.53 ± 0.11c	NS	6.46 ± 6.52	4.06 ± 2.83	2.13 ± 2.23
V19	NS	0.96 ± 0.19	1.53 ± 0.31	0.05 ± 0.01	1.75 ± 035		nd	nd	nd	nd		nd	nd	nd	nd	–	–	–	–
∑ESTs		5.18	4.79	2.26	18.0		1.82	3.37	8.20	2.87		3.30	nd	4.68	0.53				
FURs																			
V2	**	0.50 ± 0.10b	1.10 ± 0.22b	3.65 ± 0.73a	0.72 ± 0.14b	NS	2.95 ± 0.39	1.55 ± 0.52	2.59 ± 0.31	2.09 ± 0.42	**	1.91 ± 0.32b	3.17 ± 0.36a	1.80 ± 0.45b	3.65 ± 0.73a	NS	1.49 ± 1.46	2.29 ± 0.61	2.63 ± 0.92
V21	***	0.55 ± 0.11c	19.2 ± 3.84b	47.7 ± 9.55a	22.3 ± 4.46b	***	37.7 ± 12.3a	7.19 ± 1.44c	45.1 ± 9.01a	13.7 ± 2.74b	NS	14.2 ± 2.83d	79.0 ± 15.8a	25.7 ± 5.13c	55.3 ± 11.1b	NS	22.5 ± 19.4	25.9 ± 18.3	43.5 ± 29.3
∑FURs		1.05	20.3	51.4	23.0		40.7	8.74	47.7	15.8		16.1	82.2	27.5	59.0				
KETs																			
V5	***	0.46 ± 0.09b	0.35 ± 0.07b	Nd	15.5 ± 3.10a	*	nd	2.78 ± 0.56a	nd	nd	NS	nd	1.00	nd	nd	–	–	–	–
V9	***	0.99 ± 0.20b	52.8 ± 10.6a	0.86 ± 0.17b	0.65 ± 0.13b	***	2.00 ± 1.77c	14.5 ± 2.50a	4.00 ± 0.80b	13.8 ± 2.37a	*	0.41 ± 0.08b	nd	0.41 ± 0.08b	1.61 ± 0.32a	NS	13.8 ± 26.0	7.57 ± 5.38	0.61 ± 0.69
V22	*	2.46 ± 0.49b	nd	Nd	5.67 ± 1.13a	**	5.47 ± 0.94a	nd	3.00 ± 0.60b	0.94 ± 0.19bc		nd	nd	nd	nd		2.03 ± 2.69	2.35 ± 2.43	–
∑KETs		3.91	53.2	0.86	21.8		7.47	17.3	7.00	14.7		0.41	1	0.41	1.61				
TEs																			
V6	*	3.86 ± 0.77a	0.60 ± 0.12b	1.27 ± 0.25b	0.89 ± 0.18b		nd	nd	nd	nd		nd	nd	nd	nd	–	–	–	–
V7	*	1.21 ± 0.24b	0.23 ± 0.05b	3.46 ± 0.69a	1.04 ± 0.21b		nd	nd	nd	nd	***	3.58 ± 1.56a	2.38 ± 0.10ab	0.50 ± 0.34b	1.61 ± 0.32bc	NS	1.48 ± 1.38	–	2.02 ± 1.30
V10		nd	nd	Nd	nd	**	8.60 ± 2.61a	0.47 ± 0.09b	2.86 ± 0.57ab	1.00 ± 0.20b	*	2.89 ± 1.06a	1.90 ± 0.27ab	0.70 ± 0.14b	1.64 ± 0.33ab	NS	–	3.22 ± 3.70	1.78 ± 0.90
V13	NS	1.91 ± 0.38	1.35 ± 0.27	0.71 ± 0.14	2.26 ± 0.45	*	7.10 ± 1.68a	2.04 ± 0.41b	3.13 ± 0.63b	3.08 ± 0.62b		nd	nd	nd	nd	NS	1.56 ± 0.68	3.84 ± 2.23	–
V23	**	2.14 ± 0.43b	0.60 ± 0.12c	4.48 ± 0.90a	0.66 ± 0.13c		nd	nd	nd	nd		nd	nd	nd	nd	–	–	–	–
∑TEs		9.12	2.78	9.92	4.85		15.7	2.51	5.99	4.08		6.47	4.28	1.20	3.25				
TOs																			
V16		nd	nd	Nd	nd	NS	0.73 ± 0.89	2.67 ± 0.36	2.58 ± 0.34	2.26 ± 0.31		nd	nd	nd	nd	–	–	–	–
V17	**	4.00 ± 0.80a	1.07 ± 0.21b	1.05 ± 0.21b	2.03 ± 0.41b	NS	0.40 ± 0.49	0.73 ± 0.15	1.00 ± 0.20	0.49 ± 0.10	***	0.83 ± 0.17bc	1.45 ± 0.20b	35.1 ± 7.01a	2.14 ± 0.43b	NS	2.04 ± 1.39	0.66 ± 0.27	9.87 ± 16.8
V20	**	1.28 ± 0.26c	4.99 ± 1.00b	8.50 ± 1.70a	5.65 ± 1.13ab		nd	nd	nd	nd		nd	nd	nd	nd	–	–	–	–
∑TOs		5.28	6.06	9.55	7.68		1.13	3.4	3.58	2.75		0.83	1.45	35.1	2.14				

Note: Alcohols (ALCs): 3-hexen-1-ol (V3), 1-hexanol (V4), 1-octanol (V14), and 2-ethyl-1-hexanol (V15); Aldehydes (ALDs, total aldehydes): hexanal (V1), 2-heptenal (V8), octanal (V11), and nonanal (V18); Esters (ESTs): hexyl acetate (V12), and ethyl octanoate (V19); Furans (FURs): furfural (V2), and 5-HMF (V21); Ketones (KETs): 3-heptanone (V5), 3-octanone (V9), and β-damascenone (V22); Terpenes (TEs): α-pinene (V6), β-pinene (V7), α-terpinene (V10), limonene (V13) and α-gurjunene (V23); Terpenoids (TOs): linalool oxide (V16), linalool (V17), and terpinen-4-ol (V20); F: fig; J: jujube; Q: quinces; ^†^ NS = not significant at *p* < 0.05; *, **, and ***, significant at *p* < 0.05, 0.01, and 0.001, respectively. ^‡^ Values (mean of 3 replications) followed by the same letter, within the same row for the same fruit purée, were not significantly different (*p* < 0.05), according to Tukey’s least significant difference test; ^ϒ^nd: not detected, meaning below the quantification limit.

**Table 4:** Descriptive sensory analysis of smoothies prepared by blending pomegranate juice with fig, jujube, or quince purée.

ANOVA (AN) ^†^		Fig Smoothies				AN	Jujube Smoothies				AN	Quinces Smoothies				AN Purée Type			
		40F:60 PM	60F:40 PM	40F:60PW	60F:40PW		40J:60 PM	60J:40 PM	40J:60PW	60J:40PW		40Q:60 PM	60Q:40 PM	40Q:60PW	60Q:40PW		F	J	Q
Odor																			
Pomegranate	***	3.5 ± 0.5b ^‡^	2.8 ± 0.7b	5.4 ± 0.7a	4.9 ± 1.1a	NS	2.3 ± 1.0	1.0 ± 0.4	1.6 ± 0.5	2.0 ± 0.7	NS	1.9 ± 1.5	1.1 ± 0.6	2.3 ± 1.3	2.1 ± 1.1	**	4.2 ± 1.2a	1.7 ± 0.5b	1.9 ± 0.5b
Fig	***	7.0 ± 0.1a	7.2 ± 0.5a	5.6 ± 0.5c	6.3 ± 0.8b											***	6.5 ± 0.7a	0.0 ± 0.0b	0.0 ± 0.0b
Jujube						NS	4.6 ± 0.9	3.8 ± 0.5	2.8 ± 1.0	3.5 ± 1.9						***	0.0 ± 0.0b	3.7 ± 0.8a	0.0 ± 0.0b
Quince											***	7.0 ± 0.0ab	8.6 ± 0.5a	5.8 ± 0.5b	8.0 ± 1.1ab	***	0.0 ± 0.0b	0.0 ± 0.0b	7.4 ± 1.3a
Apple	NS	1.5 ± 0.5	0.9 ± 0.7	1.3 ± 0.6	1.5 ± 0.4	NS	2.3 ± 1.2	2.3 ± 1.2	1.9 ± 0.9	3.0 ± 0.7	*	2.5 ± 0.6ab	3.1 ± 1.0a	1.5 ± 0.6b	3.0 ± 0.7ab	**	1.3 ± 0.3b	2.4 ± 0.5a	2.5 ± 0.7a
Pear	*	4.7 ± 0.5a	3.4 ± 1.3ab	2.9 ± 0.8b	2.8 ± 1.5b	*	7.4 ± 0.5a	6.1 ± 0.6ab	5.1 ± 1.0b	5.6 ± 1.5ab	NS	2.6 ± 0.8	3.6 ± 1.1	2.4 ± 1.1	2.6 ± 1.3	***	3.5 ± 0.8b	6.1 ± 1.0a	2.8 ± 0.6b
Grape must	*	5.8 ± 0.8a	3.3 ± 1.9b	4.4 ± 1.4ab	2.9 ± 1.7b	*	4.6 ± 1.5ab	3.0 ± 1.4b	2.9 ± 1.3b	2.6 ± 1.1a	NS	1.0 ± 0.4	1.5 ± 1.5	1.0 ± 0.9	0.8 ± 0.6	**	4.1 ± 1.3a	3.3 ± 0.9a	1.1 ± 0.3b
Cranberry	NS	0.7 ± 0.6	0.4 ± 0.4	1.7 ± 0.3	1.2 ± 0.8	NS	0.5 ± 0.4	0.3 ± 0.3	0.3 ± 0.3	1.3 ± 0.5	NS	0.4 ± 0.3	0.6 ± 0.6	1.3 ± 1.2	1.4 ± 0.9	NS	1.0 ± 0.6	0.6 ± 0.5	0.9 ± 0.5
Floral	NS	0.4 ± 0.5	0.3 ± 0.4	0.6 ± 0.5	0.5 ± 0.4	*	2.1 ± 0.3a	1.0 ± 0.7ab	1.0 ± 0.8ab	0.6 ± 0.5b	NS	2.0 ± 0.4	2.5 ± 1.2	1.8 ± 1.2	1.9 ± 1.4	**	0.5 ± 0.1b	1.2 ± 0.6b	2.1 ± 0.3a
Green	NS	1.4 ± 0.6	0.8 ± 0.5	1.8 ± 0.9	1.4 ± 1.0	***	0.6 ± 0.3b	0.4 ± 0.3b	0.6 ± 0.3b	1.6 ± 0.3a	NS	0.8 ± 0.3	0.6 ± 0.5	0.8 ± 0.9	1.0 ± 1.1	NS	1.4 ± 0.4	0.8 ± 0.6	0.8 ± 0.2
Basic tastes																			
Sweet	***	7.5 ± 0.8a	4.3 ± 0.8c	6.8 ± 0.4ab	6.3 ± 1.4b	***	6.4 ± 0.3a	4.1 ± 0.9b	5.3 ± 0.3ab	4.5 ± 0.6b	NS	4.0 ± 0.7	4.4 ± 1.4	3.6 ± 1.1	4.0 ± 0.7	**	6.2 ± 1.4a	5.1 ± 1.0ab	4.0 ± 0.3b
Sour	***	1.1 ± 0.7c	0.9 ± 0.6c	4.8 ± 0.4a	3.8 ± 1.3b	***	1.9 ± 0.3b	2.1 ± 1.0b	5.6 ± 0.5a	5.0 ± 0.0a	***	1.8 ± 0.9b	2.1 ± 1.4b	5.9 ± 1.3a	5.3ab	NS	2.7 ± 1.9	3.7 ± 1.9	3.8 ± 2.1
Bitter	NS	0.5 ± 0.5	0.5 ± 0.4	0.5 ± 0.5	0.9 ± 0.7	NS	1.0 ± 0.4	1.1 ± 0.3	0.9 ± 0.9	0.9 ± 0.5	NS	0.8 ± 0.6	1.0 ± 0.7	1.1 ± 0.9	0.6 ± 1.3	*	0.6 ± 0.2b	0.8 ± 0.1ab	1.0 ± 0.2a
Astringent	NS	0.3 ± 0.4	0.3 ± 0.4	0.7 ± 0.6	0.7 ± 0.5	NS	1.0 ± 0.6	2.1 ± 0.8	1.1 ± 0.5	1.8 ± 1.0	NS	1.3 ± 1.2	1.5 ± 1.2	1.5 ± 0.4	1.1 ± 0.8	**	0.5 ± 0.2b	1.5 ± 0.5a	1.4 ± 0.2a
Flavor																			
Pomegranate	***	2.4 ± 0.9c	1.6 ± 0.6c	5.8 ± 0.9a	4.7 ± 1.2b	***	4.4 ± 0.5b	2.3 ± 0.5c	6.1 ± 0.6a	2.9 ± 0.9c	**	2.6 ± 0.9ab	1.5 ± 0.7b	3.9 ± 0.6a	3.0 ± 0.9ab	NS	3.6 ± 1.9	3.9 ± 1.7	2.8 ± 1.0
Fig	*	5.7 ± 0.6a	3.9 ± 0.8b	4.2 ± 1.0b	4.9 ± 1.2ab											***	4.7 ± 0.8a	0.0 ± 0.0b	0.0 ± 0.0b
Jujube						*	4.1 ± 0.3b	4.5 ± 0.6ab	5.5 ± 0.4a	4.8 ± 1.0ab						***	0.0 ± 0.0b	4.7 ± 0.6a	0.0 ± 0.0b
Quince											*	4.9 ± 1.7b	6.3 ± 1.3a	4.4 ± 1.1b	6.6 ± 1.4a	***	0.0 ± 0.0b	0.0 ± 0.0b	5.6 ± 1.1a
Apple	*	1.1 ± 0.8b	0.7 ± 0.4b	2.6 ± 1.2a	1.8 ± 1.8ab	NS	3.3 ± 1.3	2.9 ± 1.5	2.9 ± 1.9	2.8 ± 1.0	NS	2.8 ± 1.7	4.0 ± 1.1	2.4 ± 1.1	3.4 ± 1.0	**	1.6 ± 0.8b	3.0 ± 0.2a	3.2 ± 0.7a
Pear	NS	2.9 ± 1.4	1.7 ± 0.8	1.8 ± 1.0	1.9 ± 1.4	NS	6.6 ± 0.5	5.0 ± 1.4	4.8 ± 0.5	5.0 ± 1.1	NS	3.1 ± 1.8	3.4 ± 1.1	2.6 ± 1.5	3.1 ± 1.0	***	2.1 ± 0.6b	5.4 ± 0.9a	3.1 ± 0.3b
Grape must	**	4.8 ± 1.7a	1.5 ± 0.8c	3.6 ± 1.7ab	2.3 ± 1.2bc	NS	2.8 ± 1.5	1.6 ± 1.1	2.5 ± 1.3	2.4 ± 1.5	NS	1.4 ± 0.8	1.6 ± 1.6	1.6 ± 1.1	1.6 ± 2.0	NS	3.1 ± 1.4	2.3 ± 0.5	1.6 ± 0.1
Cranberry	***	0.3 ± 0.4c	0.3 ± 0.4c	3.8 ± 1.9a	2.2 ± 1.6b	***	0.6 ± 0.5b	0.3 ± 0.3b	3.5 ± 0.4a	1.5 ± 1.5b	NS	0.5 ± 0.4	0.4 ± 0.5	1.8 ± 1.2	1.5 ± 1.1	NS	1.7 ± 1.5	1.5 ± 1.4	1.1 ± 0.7
Floral	NS	0.3 ± 0.3	0.2 ± 0.3	0.3 ± 0.9	0.3 ± 0.3	NS	1.3 ± 0.6	0.6 ± 0.6	1.0 ± 0.7	0.9 ± 0.5	NS	1.1 ± 0.6	1.1 ± 0.9	1.1 ± 0.8	0.9 ± 0.8	***	0.3 ± 0.1b	1.0 ± 0.3a	1.1 ± 0.1a
Green	***	0.9 ± 0.4b	0.6 ± 0.4b	2.8 ± 1.3a	0.9 ± 0.7b	NS	0.9 ± 0.5	0.8 ± 0.9	2.0 ± 0.7	2.0 ± 1.1	NS	1.8 ± 1.0	1.4 ± 0.9	2.3 ± 0.9	1.3 ± 1.8	NS	1.3 ± 1.0	1.4 ± 0.7	1.7 ± 0.4
Texture																			
Fiberness	NS	0.0 ± 0.0	0.1 ± 0.2	0.0 ± 0.0	0.1 ± 0.2	***	9.0 ± 0.0ab	9.9 ± 0.3a	8.6 ± 0.5b	9.1 ± 1.0ab	NS	3.9 ± 1.9	6.4 ± 3.1	4.9 ± 3.2	5.4 ± 3.1	***	0.1 ± 0.0c	9.2 ± 0.5a	5.2 ± 1.0b
Consistency	***	1.2 ± 0.4c	8.9 ± 0.2a	1.3 ± 0.4c	3.8 ± 1.2b	***	1.0 ± 0.0d	6.3 ± 0.3a	2.0 ± 0.7c	5.0 ± 0.0b	NS	2.8 ± 0.3c	6.8 ± 0.3b	2.5 ± 0.4c	8.1 ± 0.3a	NS	3.8 ± 3.5	3.6 ± 2.5	5.1 ± 2.8
Defect																			
Cooked (odor)	*	1.1 ± 1.5ab	0.0 ± 0.0b	1.8 ± 1.3a	0.0 ± 0.0b	NS	1.4 ± 0.6	1.5 ± 0.9	0.4 ± 0.5	0.8 ± 0.6	***	0.3 ± 0.3	0.6 ± 0.8	0.3 ± 0.3	0.1 ± 0.3	NS	0.7 ± 0.6	1.0 ± 0.5	0.3 ± 0.2
Cooked (flavor)	NS	0.8 ± 0.9	0.0 ± 0.0	0.8 ± 1.0	0.0 ± 0.0	NS	0.6 ± 0.9	0.6 ± 0.3	0.4 ± 0.5	0.4 ± 0.5	NS	1.4 ± 0.9	0.6 ± 0.8	0.1 ± 0.3	0.3 ± 0.3	NS	0.4 ± 0.3	0.5 ± 0.1	0.6 ± 0.5

Note: ^†^ NS = not significant at *p* < 0.05; *, **, and ***, significant at *p* < 0.05, 0.01, and 0.001, respectively. ^‡^ Values (mean of 3 replications) followed by the same letter, within the same row for the same fruit purée, were not significantly different (*p* < 0.05), according to Tukey’s least significant difference test.

**Table 5:** Pearson correlations between volatile compounds and sensory descriptive attributes in smoothies prepared by blending pomegranate juice (Pom) with fig (F), jujube (J), or quince (Q) purée.

R	Odor Attributes										Basic Taste			Flavor Attributes					
	Pom	F	J	Q	Apple	Pear	Grape	Floral	Green	Sweet	Bitter	Astringency	F	J	Q	Apple	Pear	Grape	Floral
V1																			
V2																			
V3																			
V4																			
V5																			
V6																			
V7																			
V8																			
V9																			
V10																			
V11																			
V12																			
V13																			
V14																			
V15																			
V16																			
V17																			
V18																			
V19																			
V20																			
V21																			
V22																			
V23																			

Green: R > 0.6, *p*-value < 0.05; Pink: R < −0.6, *p*-value < 0.05; Orange: 0.0 < R < 0.6, NS; and Blue: −0.6 < R < 0.0, NS. Cranberry aroma, cranberry odor, green aroma, sour and pomegranate do not appear in this table since no significant correlation was found.
